# Modelling of Thyroid Peroxidase Reveals Insights into Its Enzyme Function and Autoantigenicity

**DOI:** 10.1371/journal.pone.0142615

**Published:** 2015-12-01

**Authors:** Sarah N. Le, Benjamin T. Porebski, Julia McCoey, James Fodor, Blake Riley, Marlena Godlewska, Monika Góra, Barbara Czarnocka, J Paul Banga, David E. Hoke, Itamar Kass, Ashley M. Buckle

**Affiliations:** 1 Biomedicine Discovery Institute and Department of Biochemistry and Molecular Biology, Monash University, Clayton, Australia; 2 The Centre of Postgraduate Medical Education, Department of Biochemistry and Molecular Biology, Warsaw, Poland; 3 Institute of Biochemistry and Biophysics PAS, Department of Genetics, Warsaw, Poland; 4 King's College London School of Medicine, Division of Diabetes and Nutrition Sciences, London, United Kingdom; Russian Academy of Sciences, Institute for Biological Instrumentation, RUSSIAN FEDERATION

## Abstract

Thyroid peroxidase (TPO) catalyses the biosynthesis of thyroid hormones and is a major autoantigen in Hashimoto’s disease—the most common organ-specific autoimmune disease. Epitope mapping studies have shown that the autoimmune response to TPO is directed mainly at two surface regions on the molecule: immunodominant regions A and B (IDR-A, and IDR-B). TPO has been a major target for structural studies for over 20 years; however, to date, the structure of TPO remains to be determined. We have used a molecular modelling approach to investigate plausible modes of TPO structure and dimer organisation. Sequence features of the C-terminus are consistent with a coiled-coil dimerization motif that most likely anchors the TPO dimer in the apical membrane of thyroid follicular cells. Two contrasting models of TPO were produced, differing in the orientation and exposure of their active sites relative to the membrane. Both models are equally plausible based upon the known enzymatic function of TPO. The “trans” model places IDR-B on the membrane-facing side of the myeloperoxidase (MPO)-like domain, potentially hindering access of autoantibodies, necessitating considerable conformational change, and perhaps even dissociation of the dimer into monomers. IDR-A spans MPO- and CCP-like domains and is relatively fragmented compared to IDR-B, therefore most likely requiring domain rearrangements in order to coalesce into one compact epitope. Less epitope fragmentation and higher solvent accessibility of the “cis” model favours it slightly over the “trans” model. Here, IDR-B clusters towards the surface of the MPO-like domain facing the thyroid follicular lumen preventing steric hindrance of autoantibodies. However, conformational rearrangements may still be necessary to allow full engagement with autoantibodies, with IDR-B on both models being close to the dimer interface. Taken together, the modelling highlights the need to consider the oligomeric state of TPO, its conformational properties, and its proximity to the membrane, when interpreting epitope-mapping data.

## Introduction

Thyroid peroxidase (TPO) plays an essential role in thyroid hormone synthesis, catalysing the iodination of tyrosines on thyroglobulin as well as the synthesis of triiodothyronine and thyroxine [1]. TPO is a major autoantigen in autoimmune thyroid diseases (AITDs), encompassing Hashimoto’s thyroiditis and Graves’ disease. Many AITD patients test positive for autoantibodies against thyroid proteins, in particular TPO (reviewed in [2]). There is evidence that antibodies against TPO are responsible for the autoimmune destruction of thyrocytes [3], either by fixing complement or through cell mediated cytotoxicity [4]. However, antibodies against other thyroid proteins, notably thyroid stimulating hormone receptor (TSHR), may be more important in some AITDs [5], and antibody-mediated cytotoxicity may be a secondary mechanism to thyroid destruction [3, 6]. It has also been shown that the complement pathway may be directly activated by component C4 binding to TPO itself [7].

**Fig 1:**
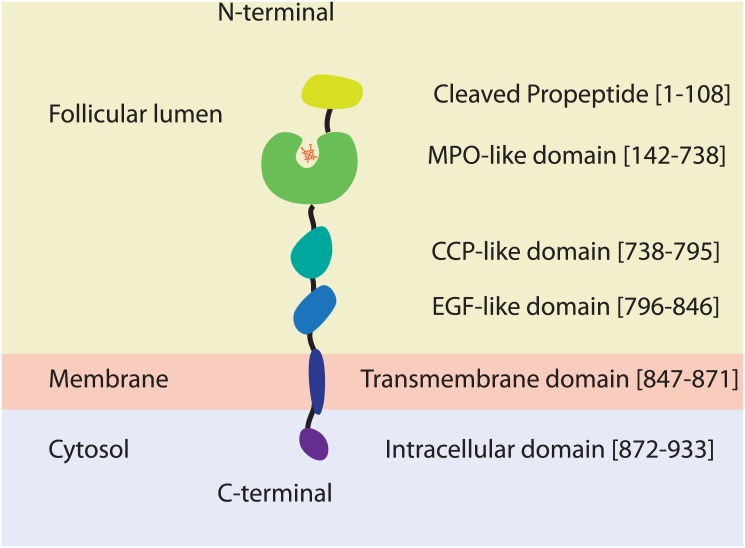
Schematic showing domain organization of TPO, as determined from sequence analysis. The protein sequence is coding an N-terminal propeptide domain (yellow), a MPO-like domain (green) with a catalytic heme (red) that is connected to a CCP-like (Shamrock green), and an EGF-like (cyan-blue) domains, an anchoring trans-membrane domain (dark-blue) and an intracellular domain (dark-magenta).

TPO is a 933-residue transmembrane homodimer [8–10]. It is a largely extracellular protein that is likely anchored to the apical membrane of thyroid follicle cells by a putative transmembrane region at its C-terminal end (residues 847–871) [11, 12]. The N-terminal propeptide sequence (residues 1–108) is cleaved in the mature protein [13, 14]. Three regions in the TPO ectodomain (residues 109–846) exhibit a high degree of sequence similarity to domains of known three-dimensional structure: myeloperoxidase (MPO)-like domain (residues 142–738), complement control protein (CCP)-like domain (residues 740–795), and epidermal growth factor (EGF)-like domain (residues 796–846) [15] (Fig 1).

The closest known homologues of TPO are lactoperoxidase (LPO), myeloperoxidase (MPO), and eosinophil peroxidase (EPO), sharing 48%, 47% and 47% sequence identity with TPO’s MPO-like domain, respectively. X-ray crystal structures have been determined for MPO and LPO [16, 17]. Although low-resolution crystals of TPO were reported over 15 years ago [18, 19], its structure remains to be determined.

Over the last 20 years there have been considerable efforts in mapping the regions of TPO that are responsible for its autoantigenicity (see [4] and [20] for a review). Epitope mapping studies with patient autoantibodies, murine monoclonal antibodies and recombinant human anti-TPO antigen-binding (Fab) fragments reveal that the autoimmune response to TPO is directed mainly at two surface regions on the molecule: immunodominant region A (IDR-A) and immunodominant region B (IDR-B) [21–24]. The majority of residues that compose these two epitopes are found on the MPO-like domain, but there is evidence to suggest that residues from the neighboring CCP-like domain are also involved [24, 25]. The structural interpretation of these data is limited by the absence of an experimentally determined structure, so the structural basis of TPO autoimmunity remains poorly understood.

The X-ray crystal structure determination of human myeloperoxidase (MPO) in 1995 [26] provided a platform for investigating the structural basis of TPO autoantigenicity, due to the high sequence similarity between MPO and the MPO-like domain in TPO. The structure of MPO was used to design and interpret mutagenesis experiments aimed at probing the antigenicity of TPO [27], but was limited to the MPO-like domain only. The vicinity to and orientation with respect to the membrane was not considered. Subsequent identification of an autoepitope encompassing the C-terminal region of TPO prompted the modelling of the EGF-like and CCP-like domains [28]. The study demonstrated the need to consider the structure of the entire extracellular portion of TPO, not only the MPO-like domain, when investigating the autoantigenicity of TPO. Our earlier study mapped epitope data onto a model of all the extracellular domains of TPO [24], but did not consider interactions with the membrane.

In this work we have used a molecular modelling approach combined with molecular dynamics (MD) simulations to investigate plausible models of TPO dimer organisation, and how they may be used to gain insight into the nature of its autoepitopes. This study highlights the need to consider TPO’s oligomeric state, conformational properties, and proximity to the membrane, in the context of its enzymatic function when interpreting antigenicity data.

## Materials and Methods

### Modelling

Template sequences were selected by querying the TPO protein sequence against the Protein Databank (PDB) using the NCBI BLAST web server (http://blast.ncbi.nlm.nih.gov/Blast.cgi). The MPO-like, EGF-like, and CCP-like domains were modelled using for templates Protein Data Bank (PDB) entries 1CXP [16], 1EMO [29], and 1VVD [30], respectively. Specifically, we used the following residues for modelling: MPO-like domain (residues 142–738 modelled using residues 167–744 from template 1CXP, 47% sequence identity), CCP-like domain (residues 740–795 modelled using residues 147–202 from template 1VVD, 35% sequence identity), and EGF-like domain (residues 798–839 modelled using residues 2162–2205 from template 1EMO, 39% sequence identity). The cholinesterase-like (ChEL) domain of thyroglobulin was modelled using the crystal structure of recombinant human acetylcholinesterase in the apo state (PDB ID: 4EY4) [31] as a template. Transmembrane regions of TPO were identified using the membrane protein prediction server TMHMM [32], which found a transmembrane helix immediately after the EGF-like domain. A putative helix-helix interaction motif was identified as reported [33]. For the generation of the transmembrane helices dimer, the structure of a GxxxG motif dimer (PDB ID: 2L2T)[34] was used as a template. 200 homology models of human TPO (residues 142–880) were built using MODELLER v9.12 [35] and sorted by their Discrete Optimized Protein Energy (DOPE) score. The inter-dimer disulfide bridge (residues 153 in 1CXP) was modelled in the TPO dimer model, as was the iron-protoporphyrin IX (heme) group. Symmetry was maintained using MODELLER symmetry restraints between the two chains. Model plausibility was assessed by visual inspection of domain juxtaposition, and by determining the quality of the models using three separate assessments of model quality: (1) VERIFY 3D [36], which determines the compatibility of an atomic model with its own amino acid sequence by assigning a structural class based on its location and environment (alpha, beta, loop, polar, nonpolar etc.) and comparing the results to good structures. All models pass the VERIFY 3D test; (2) MolProbity [37], a widely used method (used by the PDB, for example) to asses the stereochemical quality of structures; (3) QMEAN Server for model quality estimation [38] (http://swissmodel.expasy.org/qmean), a method for the estimation of the absolute quality of individual protein structure models which is independent of protein size.

### Preparation of MD systems

The initial coordinates for TPO isoforms 1 and 4 were modelled as described above. Each isoform was then embedded in 1,2-dimyristoyl-*sn*-glycero-3-phosphocholine (DMPC) membrane and solvated in water using CHARMM-GUI [39]. DMPC lipids are widely used in studies that employ MD because they are well parameterized and simulation outcome is in good agreement with experimental data regarding physical properties such as area per lipid, bilayer thickness and electron density profile [40, 41]. Water molecules were then randomly replaced with Ca^2+^, necessary for TPO activity, and Na^+^ counter-ions, in order to neutralize any net charges in simulated systems. Simulation boxes were built such that the minimum distance between any protein atom to the box face is 20 Å. Initially, each system was subjected to 50,000 steps of energy minimization. The simulated systems were then gradually heated to 300 K over 0.2 ns, with the protein heavy-atoms harmonically constrained, under the canonical ensemble (NVT) conditions. Following this, each system was simulated with the heavy-atoms constraints gradually removed for 0.55 ns under isothermal—isobaric ensemble (NPT) conditions.

### MD simulations

MD simulations were performed using NAMD v2.9 software [42] in conjunction with the Amber ff14SB all-atom force field [43] and TIP3P water molecules [44]. DMPC force-field parameters were taken from [40]. The simulation timestep was set at 2fs, and the nonbonded cut-off length was set at 10 Å, with a pair-list cut-off set at 12 Å. All simulations were run at constant temperature (300K) and pressure (1atm) (NPT), using a Langevin damping coefficient of 5 ps^−1^. For each simulated system, periodic boundary conditions were used together with the Particle Mesh Ewald algorithm for long-range electrostatic interactions [45]. System conformations were saved every 10 ps for analysis. Each system was subjected to free simulation on a Blue Gene/Q cluster for a total of 30 ns. Simulations were performed in duplicate—each with different initial velocities—to minimize simulation biasing.

### MD analysis

Analysis was performed using GROMACS [46] and visualizations using VMD 1.9.1 [47]. Figures and movies were rendered using VMD and PyMol 1.3 [48]. Root mean square deviation (RMSD) of backbone heavy atoms with respect to their initial structure were calculated every 10 ps, after performing a least-squares fit to their initial structure.

## Results and Discussion

### Modelling the structure and architecture of the extra-cellular domains of TPO

**Fig 2:**
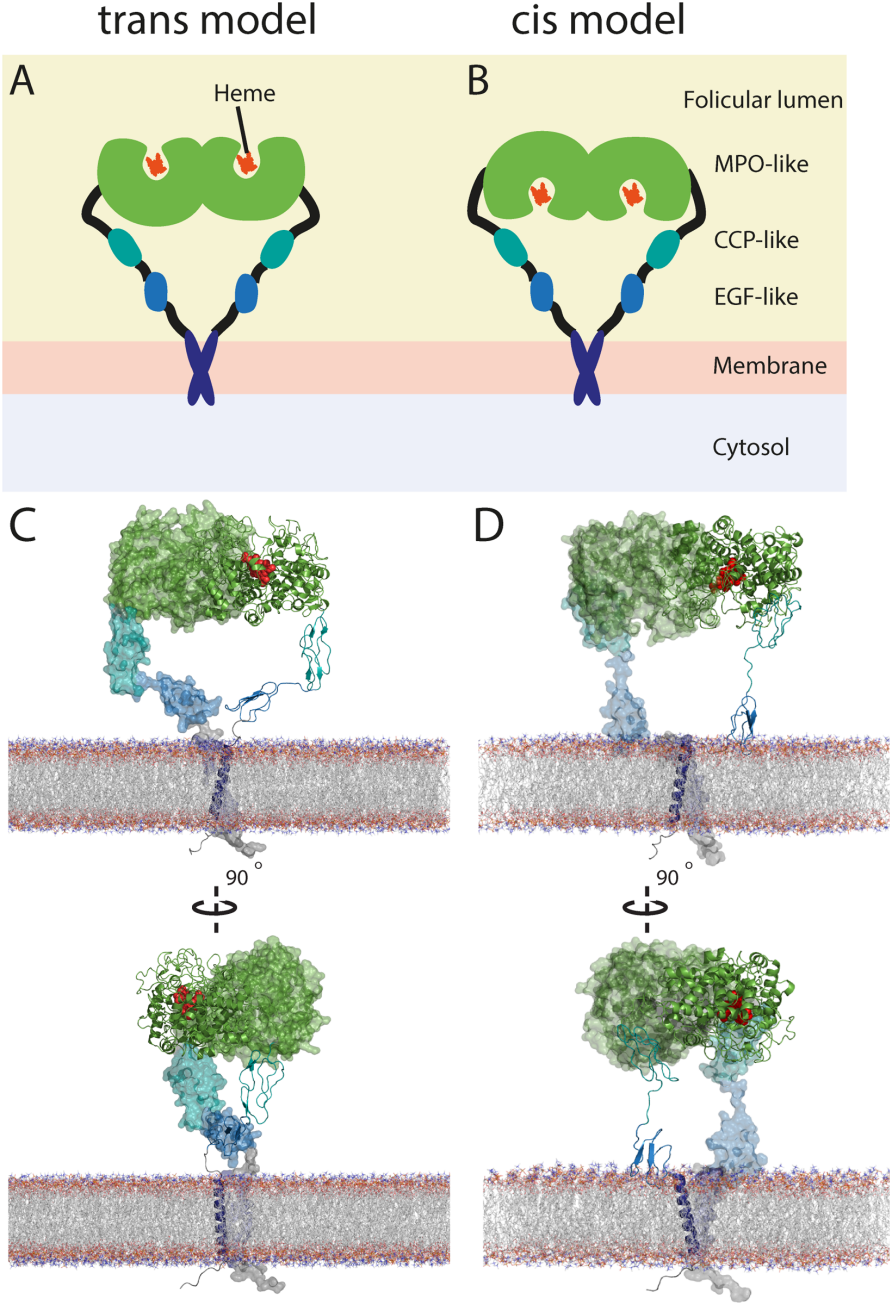
A comparison between membrane-embedded, dimeric models of TPO isoform1 in two different conformations. **(A)** Schematic of trans model with active site facing away from follicular membrane. Coloring as in Fig 1; **(B)** Cis model with active site facing towards follicular membrane; **(C)** perpendicular views of trans model; **(D)** perpendicular views of cis model. TPO represented as cartoon and space filling (subunits A and B, respectively) for clarity. Domains are coloured as follows: MPO-like domain (green), CCP-like domain (Shamrock green), EGF-like domain (cyan-blue), and TM domain (dark-blue). Catalytic heme represented as red spheres, and DMPC molecules represented as lines and coloured as CPK.

High sequence identity between human TPO and proteins with known structures enabled homology modelling of the three extracellular domains (S1 Fig). Crystal structures are available for LPO and MPO, both of which have high sequence identity to the MPO-like domain of TPO [16, 17]. However, MPO is a more reasonable template as it forms disulphide-linked dimers, whereas LPO is monomeric. TPO functions as a dimer, linked via a conserved intermolecular disulphide bond at the same location as in MPO (TPO Cys296) at the dimer interface [8, 49] [10]. Therefore, we modelled the MPO-like domain (residues 142–738) using the crystal structure of human MPO. The dimer interface in the MPO structure defines the relative orientation of the monomers in the TPO model. Unlike other known peroxidases, TPO has a transmembrane (TM) domain, as well as CCP-like and EGF-like domains linking the MPO-like domain and the TM domain (Fig 1). The CCP-like (residues 740–795) and EGF-like (residues 796–846) domains likewise have high sequence identity homologues for which structures are known (S1 Fig). Together, this enabled homology modelling of all three extracellular domains and the TM domain of TPO (Fig 2).

### Transmembrane helix association

A 25 amino-acid sequence has previously been identified as a likely transmembrane domain using hydropathy profiling [11]. Bioinformatic analysis [50] of this region suggests an α-helical conformation. Upon closer inspection of this region, we identified a transmembrane helix dimerization motif, G^860^xxxG^864^ (S1 Fig). The GxxxG motif is indicative of a dimeric coiled-coil structure of transmembrane helices [33, 51, 52]. There are two additional features that support transmembrane helix dimerization in TPO. First, the isoleucine that precedes the first glycine in the motif is frequently seen in transmembrane helices [51]. Second, there is a threonine in the 4^th^ position after the GxxxG motif (S1 Fig); GxxxGxxxT is the most represented three-residue pattern subset of the LIxxGVxxGVxxT seven-residue helix-helix dimerization motif [51, 53]. We therefore were able to incorporate this predicted transmembrane helix dimerization motif into the architecture of TPO with a high degree of confidence (Fig 2).

### TPO domain organisation and enzyme function

Although the MPO-like domain of TPO can be modelled as a dimer using the known crystallographic disulphide-linked dimer of MPO, the spatial organisation of the MPO-like domains relative to the CCP-like and EGF-like domains and membrane cannot be determined from primary sequence alone. There are several factors that allow for two alternative arrangements: the order of the domains in the TPO primary sequence, symmetry restraints, the dimerization interface inferred from the structure of MPO, and the predicted dimerization in the TM domain (Fig 2). Modelling demonstrated that the two alternative dimeric arrangements, which differ in their orientation of the MPO-like domain, were both equally plausible from a purely structural perspective. Model quality and plausibility were assessed by visual inspection of domain juxtaposition, in combination with three separate assessments of model quality (see methods and S1 Table).

Consideration of the enzymatic function of TPO should be taken into account in determining which of these two arrangements is the most likely architecture. TPO catalyses two vital reactions for thyroid synthesis: iodination of tyrosine residues in thyroglobulin, and coupling of the resulting iodotyrosines to form the thyroid hormones T_3_ and T_4_ [1]. This process is dependent upon the heme group being covalently bound to TPO [54]. The two possible models for TPO orient the cavity containing the heme facing either the thyroid follicular lumen (the “trans” model; Fig 2A and 2C) or the thyrocyte membrane (the “cis” model; Fig 2B and 2D).

In the cis model, orienting the active site of TPO towards the thyrocyte membrane (Fig 2B and 2D) would strongly prohibit access by thyroglobulin. Thyroglobulin is a 2748 residue protein, existing as a 660 kDa homodimer [55]. This is approximately three times the size of TPO. Three of the four known iodinated tyrosines that are used to form thyroid hormones on human thyroglobulin are located in its C-terminal portion, known as the carboxyl-terminal cholinesterase-like (ChEL) domain for its similarity to and proposed evolutionary origin from acetylcholinesterase [56]. We modelled a potential reaction in which the tyrosine residues in thyroglobulin would require close proximity to the active site of TPO. Specifically, we modelled a 520-residue region comprising the ChEL domain of thyroglobulin based on homology with acetylcholinesterase. This small fragment, approximately one tenth of the thyroglobulin dimer, is only just small enough to fit within the space underneath the active site in the cis model of TPO (S2 Fig). Thyroglobulin is ellipsoidal, with the smaller dimension between 110 and 145 Å [55, 57, 58]. The space underneath the active site in the cis model of TPO only allows access to shapes of up to approximately 50 Å wide (estimated by measuring the dimensions of the cavity manually). Furthermore, thyroglobulin dimerizes via its ChEL domain [59], excluding the possibility of the ChEL domain extending out from the rest of the protein to gain access to the active site of TPO. However, there is as yet no conclusive kinetic evidence that iodination occurs via formation of a TPO-bound iodinated intermediate, and the mechanism of Tg iodination may be nonspecific [60]. Therefore, both trans and cis models are equally plausible based upon enzymatic function.

Interestingly, a fully active, naturally-occurring TPO isoform exists which does not contain the EGF-like domain (termed isoform4; the full length molecule is known as isoform1) [61]. Molecular models of isoform4 can be built in both the trans and the cis conformations (S3 Fig). Taken together, modelling in the context of enzyme function produces two equally plausible forms of the TPO dimer.

### TPO models are energetically stable

Having explored the likely dimeric arrangement of TPO, we next used MD simulations in order to assess the structural viability of the trans and cis models of isoform1 and isoform4. Simulations were performed after inserting the model into a DMPC lipid bilayer. Short simulations indicate that both models represent viable conformations of the TPO dimer bound to the membrane, supporting the plausibility of the dimeric trans and cis models (S4 Fig). Small structural changes of the trans-membrane spanning helices are consistent with typical behaviour of proteins in MD simulations and also reflect inaccuracies inherent in a homology model.

### Oligomeric architecture of TPO plays role in its autoantigenicity

**Fig 3:**
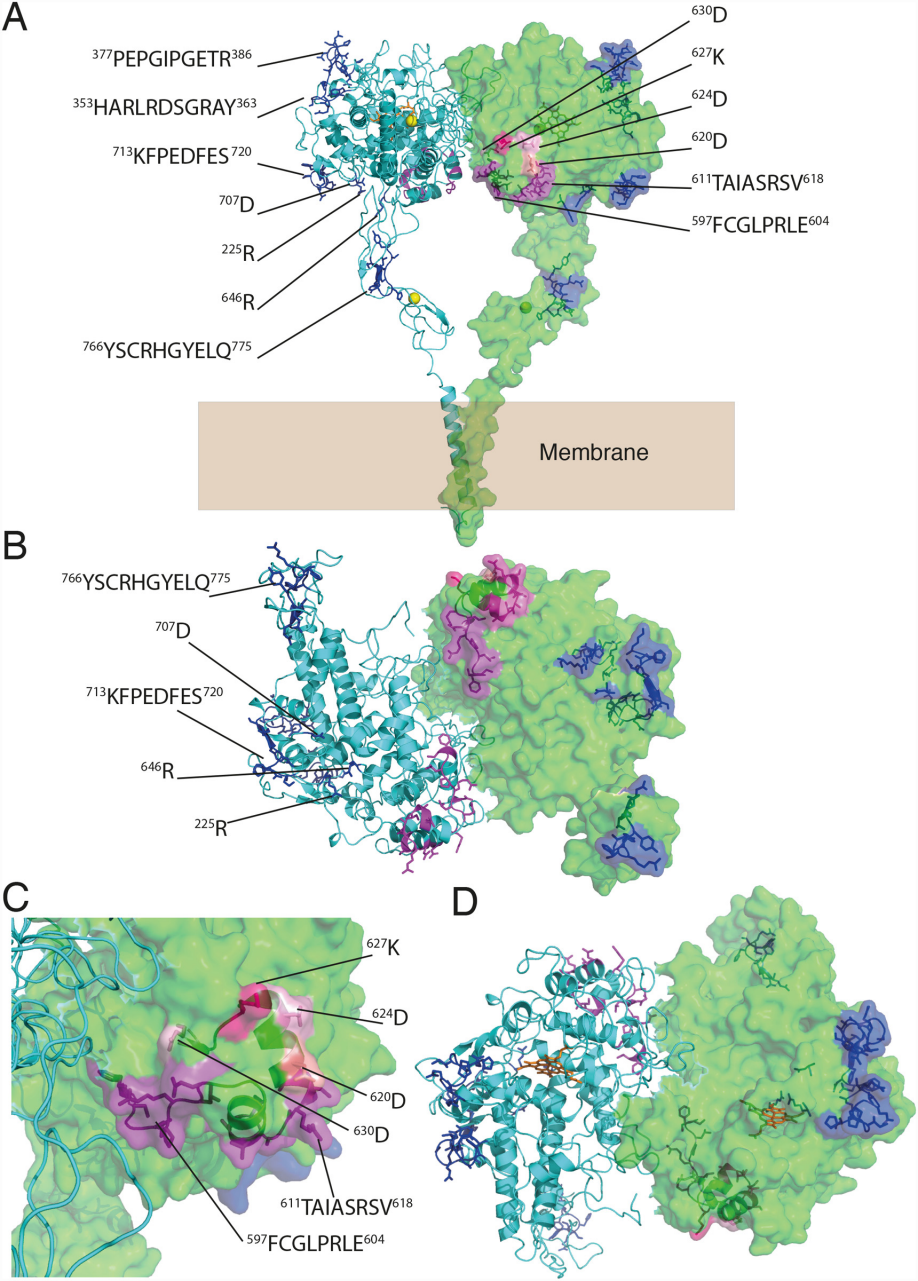
Mapping epitopes onto the trans TPO dimer. **(A)** Epitopes and residues identified in various studies as constituting immunodominant regions IDR-A (blue) and IDR-B (pink); **(B)** Membrane-facing side of MPO-like domain; **(C)** Close-up shows that much of the two immunodominant regions are on the membrane-facing surface of the MPO-like domain, particularly IDR-B; **(D)** Lumen-facing side of TPO. Hemes are shown in orange.

**Fig 4:**
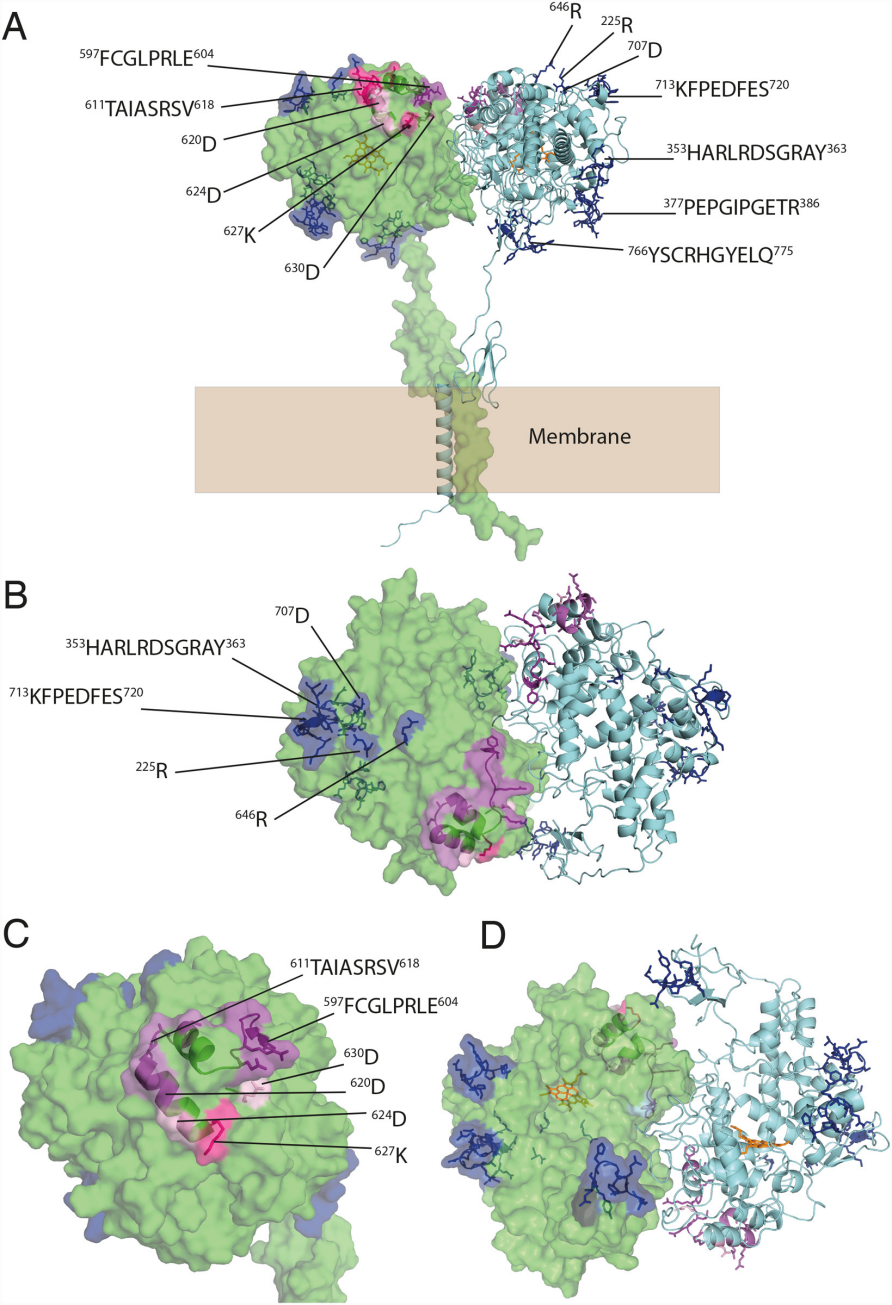
Mapping epitopes onto the cis TPO dimer. **(A)** Epitopes and residues identified in various studies as constituting immunodominant regions IDR-A (blue) and IDR-B (pink); **(B)** Lumen-facing side of MPO-like domain; **(C)** Close-up shows that much of the two immunodominant regions are on the lumen-facing surface of the MPO-like domain near the dimer interface, particularly IDR-B; **(D)** Membrane-facing side of TPO containing the heme (orange) cavity.

Having explored the structure and dynamics of TPO dimers, we next mapped the known epitopes onto both the trans and cis models. Epitope mapping studies have identified two immunodominant regions: immunodominant region A (IDR-A) and immunodominant region B (IDR-B) (S2 Table), initially defined with murine monoclonal antibodies [4, 20, 21]. These two IDRs were independently identified using human anti-TPO antibody Fabs, confusingly also named A and B, but transposed [23, 62]. We continue to use the initial nomenclature of Ruf et al. [4, 20, 21] who first identified the two IDR regions on TPO. Mapping these regions onto the trans model of the TPO dimer places IDR-B on the thyrocyte membrane-facing side of the MPO-like domain, in a closely arranged cluster near the MPO-MPO dimer interface (Fig 3). Similarly, on the cis model IDR-B clusters closely together near the dimer interface on the lumen facing side of the TPO molecule (Fig 4).

This finding suggests that engagement of antibody with IDR-B on both trans and cis models of TPO may require conformational change of TPO, and perhaps even dissociation of the dimer into monomers. The latter possibility is consistent with the identification of both monomeric and dimeric forms of autoantigenic TPO [8, 27, 63–65]. In addition, although the identification of a plausible C-terminal transmembrane dimerization motif supports a dimeric arrangement, the observation of LPO monomers as well as the absence of monomer-monomer interactions mediated via CCP-like or EGF-like domains in our models supports the plausibility of autoantibodies binding monomeric TPO. It is important to note that dimerization does not bring together IDRs from respective monomers in either cis or trans models (Figs 3 and 4). IDR-B, which is located on the underside of the MPO-like domain near the MPO-MPO interface on the trans model, shows the closest proximity of epitopes from each monomer, but they are still too separated to participate in the same antibody-binding interaction in a single epitope.

The epitopes of IDR-A, mapped onto the trans and cis model are not as closely grouped together as the epitopes of IDR-B (Figs 3 and 4). The features of our models (i.e. domains connected by flexible linkers) are consistent with some degree of inter-domain conformational flexibility, and as such, it is plausible that domain movements may consolidate the IDR-A epitope. Specifically, the CCP-like domain may be positioned such that the 766–775 epitope region on the CCP-like domain fits in place between the 377–386, 353–363 regions and the 713–720 regions on the MPO-like domain. This would connect all the epitope regions identified as part of IDR-A. To investigate this, we built several models (in both trans and cis) based on templates with this postulated arrangement of domains. Our attempts indicated that via this route, there is insufficient sequence length to cover the distance between the C-terminal end of the MPO domain and the N-terminal end of the transmembrane domain. Further, dimerization may prevent the CCP-like domain from positioning in between the IDR-A epitopes on the MPO-like domain. However, independent observations call into question the role of the 766–775 region in the IDR-A epitope. Specifically, the Y772A mutant protein failed to traffic to the cell surface, and, more importantly, reacted very poorly with both IDR-A and IDR-B-specific Fabs, IDR-B-specific polyclonal rabbit antiserum to TPO-derived peptide P14, or rabbit polyclonal antiserum to TPO [66, 67]. It has previously been shown that when both the CCP- and EGF-like domains are deleted from TPO, there is no significant impact on TPO reactivity with autoantibodies [68] and TPO-specific recombinant human Fabs [69]. Taken together, these results suggest that mutations in this region may decrease the stability of the protein, thereby indirectly weakening autoantibody binding. We have recently observed this phenomenon in the type 1 diabetes autoantigen glutamate decarboxylase 65 (GAD65) whereby epitope-mapping by mutagenesis affects both protein stability and conformational properties, giving a false-positive readout for some epitope regions (Porebski et al, unpublished).

Whereas IDR-A is solvent accessible in the trans TPO dimer (Fig 3) and therefore available to antibodies, IDR-B is somewhat occluded and may require dissociation into monomers before becoming more accessible at antibodies. In contrast, in the cis model IDR-B epitope regions cluster at the top of the MPO-like domain on TPO near the dimer interface, and are fully solvent-accessible (Fig 4). Given its likely orientation in the membrane, epitopes on the cis model face the lumen and may be more easily accessible to autoantibodies. IDR-A is surface exposed and scattered across the molecule, as in the trans model, but in much closer proximity. Calculation of electrostatic potential surfaces on both the cis and trans models reveals a heterogeneous electrostatic distribution spread across the molecular surfaces, with no discernable electrostatic pattern distinguishing epitope from non-epitope regions (S5 Fig). However, inspection of the electrostatic surface of the anti-TPO TR1.9 Fab crystal structure [70] reveals some degree of charge complementarity between the CDR regions and the 713–720 region on the IDR-A epitope of the MPO-like domain on both the cis and trans models of TPO (S6A Fig). The relative sizes of TPO and human IgG antibody are shown in S6B Fig to illustrate the concept of steric occlusion of an antibody to the TPO epitopes, and how this may be affected by the conformational properties of TPO.

Taken together, our modelling suggests that the cis dimer is slightly more favourable than the trans dimer, based on the spatial arrangement and accessibility of epitopes. However, dissociation of the dimer is likely to significantly alter the conformational properties of TPO and thus its interaction with autoantibodies. We note that the enzyme bound to the apical membrane is not accessible to the immune system; it may be that during 'reverse endocytosis' to release the thyroid hormone products in the blood, conformational changes and/or dissociation into monomers allow antibody interaction. Alternatively, it has been proposed that autoantibodies may reach TPO by transcytosis across epithelia, via a membrane Fc receptor expressed by thyrocytes [71]. Our findings invite further investigation of the relationship between antibody binding and oligomeric state, for example using mutagenesis to favour monomer formation, as well as probing potential interactions between the CCP- and MPO-like domains. The timescale of our MD simulations, and the inaccuracies inherent in a homology model, prevent further exploration of conformational change at this stage. Such an investigation would require an experimentally-determined structure as a starting point for long timescale (>100 microseconds) simulations, which are technically challenging with current state-of-the-art computational hardware.

## Conclusions

We present here refined models of a membrane-bound TPO, providing a much-needed platform for structurally interpreting epitope data and highlighting new avenues for investigation of the breakdown of immune tolerance to TPO in thyroid autoimmune disease. Both trans and cis models of dimeric TPO are equally likely based upon conformational properties as well as enzyme function, with the cis model being slightly more favorable based upon spatial positioning of epitopes. This visualization of the extensive epitope mapping data on both models of the TPO dimer provides structural insights into the basis of TPO autoantigenicity and a platform for further investigation. Both architectures may implicate significant conformational plasticity upon engagement with autoantibodies, suggesting that the oligomeric state and conformational properties of TPO play important roles in its autoantigenicity.

## Supporting Information

S1 Fig Sequence alignment of template structures to TPO sequence. Immunodominant epitopes are boxed and labelled. Domains and motifs are indicated by coloured bars.
(PNG)

S2 Fig The cis model of TPO (left) and ChEL domain of thyroglobulin (right), shown on the same scale. Two of the three tyrosines that are iodinated in ChEL are shown in blue (the remaining tyrosine is not in the construct that was crystallised). Heme groups in the buried interior of the MPO-like domains are shaded. TPO domain locations are labelled.
(PNG)

S3 Fig Model of TPO isoform4, which does not contain the EGF-like domain, in (A) trans and (B) cis conformations. One monomer is shown as cartoon, the other as molecular surface. MPO-like domains are coloured green, CCP-like domains are cyan. The transmembrane helix is blue, with one heme group shown in red.
(PNG)

S4 Fig RMSD plots for representative trajectories from MD simulations of TPO isoform1 and 4, trans (A) and cis (B) models. Average Cα backbone RMSDs (n = 1) were found to stabilize after deviating 8.5 Å for isoform1-trans, 5 Å for isoform1-cis, 4.5 Å for isoform 4-trans, and 4 Å for isoform 4-cis. RMSDs indicate that equilibrium is reached after approximately 12 ns for the trans model and 10 ns for the cis model.
(PNG)

S5 Fig Electrostatic surface of trans (A) and cis (B) models contoured at ±3 kT/e (blue is +ve, red is—ve). IDR-A is shown in pink sticks, IDR-B is shown in blue sticks and heme is shown in orange.
(PNG)

S6 Fig **(A)** Electrostatic surface potential of the TR1.9 Fab CDR and its respective IDR-A epitope on the trans and cis model. Electrostatic surfaces are contoured at ±3 kT/e (blue is +ve, red is—ve). CDR loops are also shown underneath the transparent molecular surface; **(B)** Relative size and scale of an IgG molecule and the trans model of TPO, with IDR-A coloured in blue and IDR-B coloured in magenta. This reveals the restricted space available for antibody binding.
(PNG)

S1 Table Assessment of homology model quality. ^1^Number of unfavorable all-atom steric overlaps ≥ 0.4Å per 1000 atoms. ^2^MolProbity score combines the clashscore, rotamer, and Ramachandran evaluations into a single score, normalized to be on the same scale as X-ray resolution. 100^th^ percentile is the best among structures of comparable resolution; 0^th^ percentile is the worst. ^3^Global score of the whole model reflecting the predicted model reliability ranging from 0 to 1. ^4^Estimate of the "degree of nativeness" of the structural features observed in a model by describing the likelihood that a model is of comparable quality to high-resolution experimental structures.
(DOCX)

S2 Table Epitopes and residues identified in various studies as constituting immunodominant regions A (blue, left) and B (pink, right) mapped onto the TPO model. (DOCX)
