# Supplementary figures and images for: Modelling of Thyroid Peroxidase Reveals Insights into Its Enzyme Function and Autoantigenicity
Source: PLoS One. 2015 Dec 1;10(12):e0142615. doi: 10.1371/journal.pone.0142615 (PMC4666655; doi:10.1371/journal.pone.0142615)

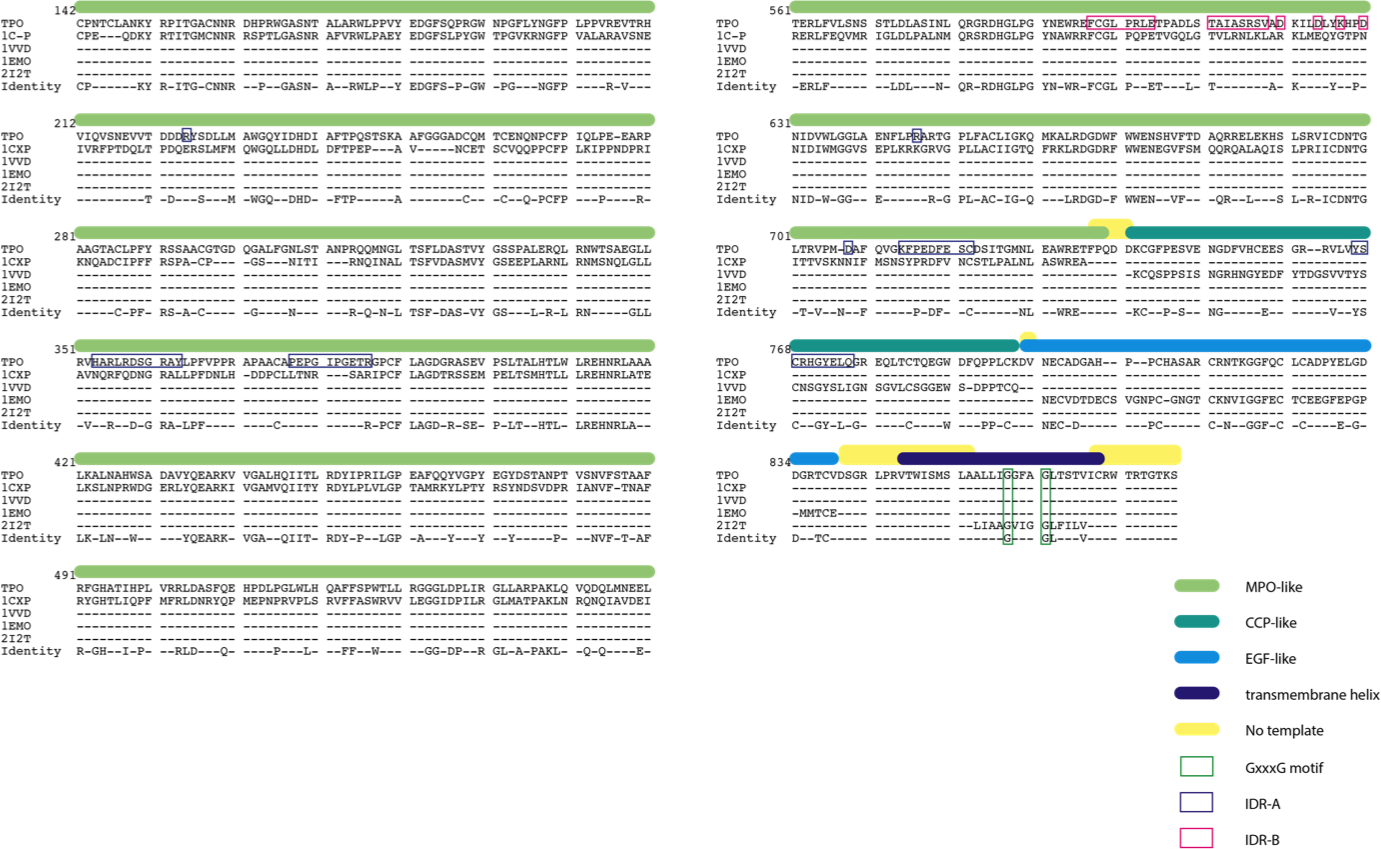

Supplement: S1 Fig — Immunodominant epitopes are boxed and labelled. Domains and motifs are indicated by coloured bars. (PNG) [file pone.0142615.s001.png]

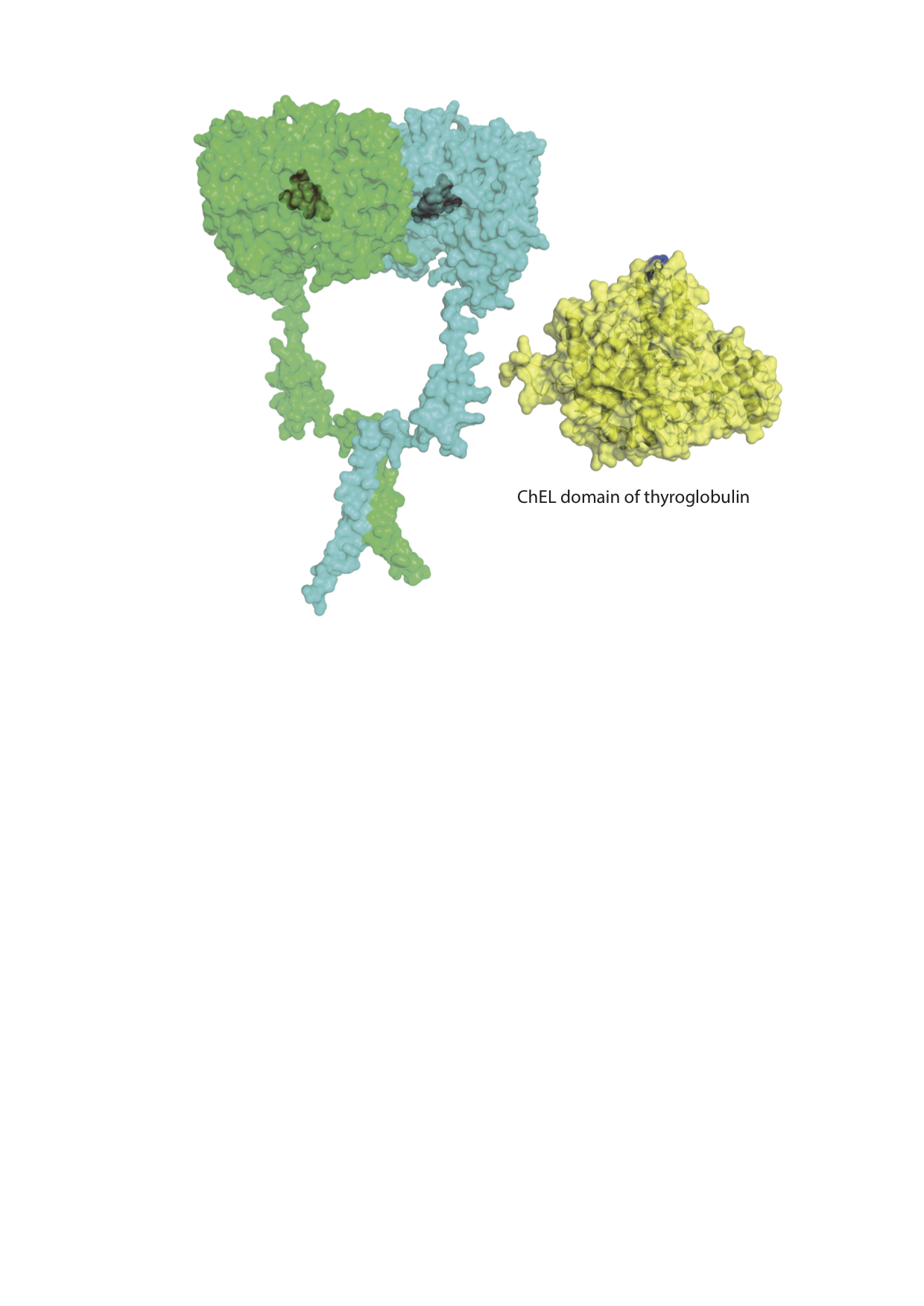

Supplement: S2 Fig — Two of the three tyrosines that are iodinated in ChEL are shown in blue (the remaining tyrosine is not in the construct that was crystallised). Heme groups in the buried interior of the MPO-like domains are shaded. TPO domain locations are labelled. (PNG) [file pone.0142615.s002.png]

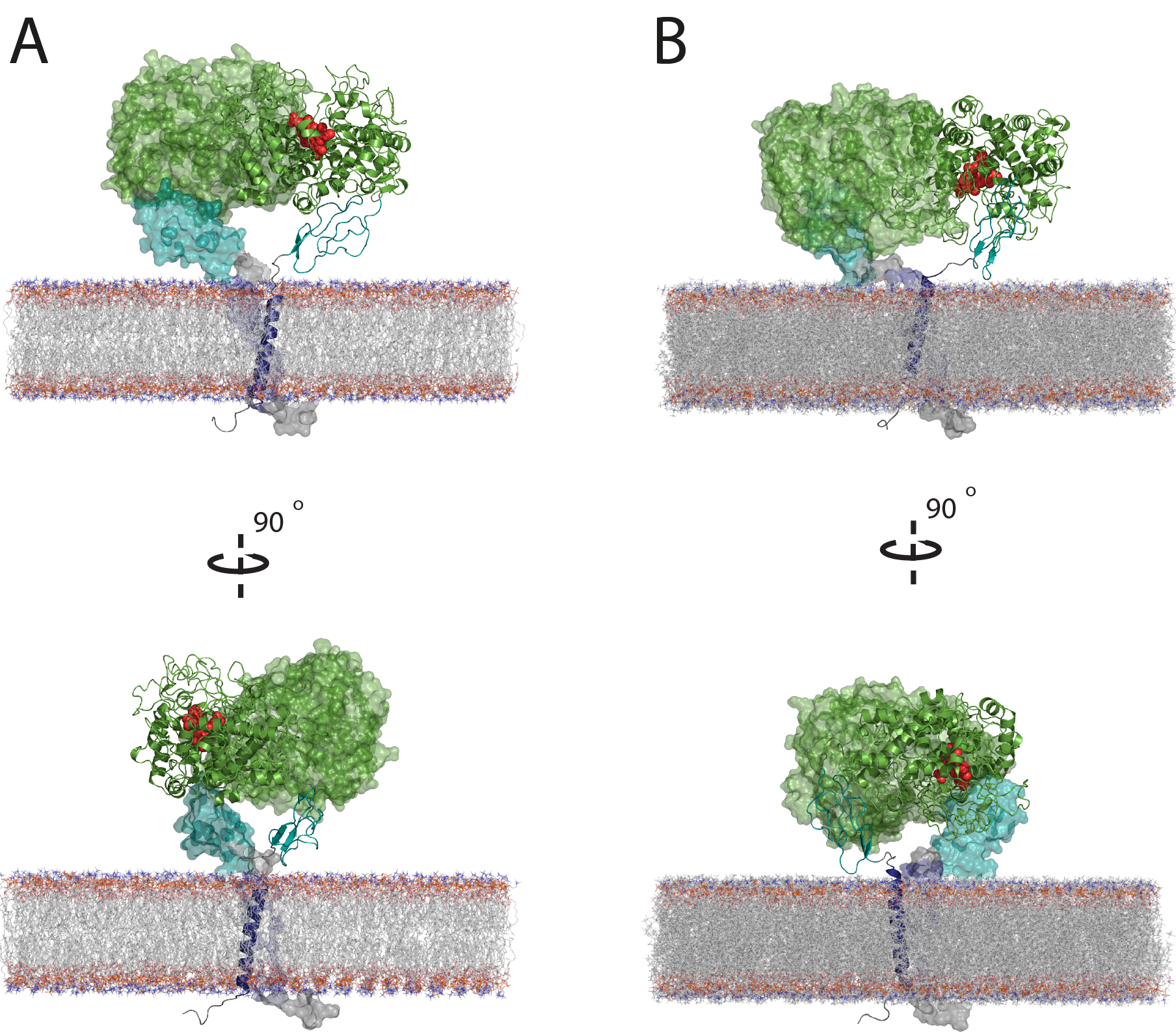

Supplement: S3 Fig — One monomer is shown as cartoon, the other as molecular surface. MPO-like domains are coloured green, CCP-like domains are cyan. The transmembrane helix is blue, with one heme group shown in red. (PNG) [file pone.0142615.s003.png]

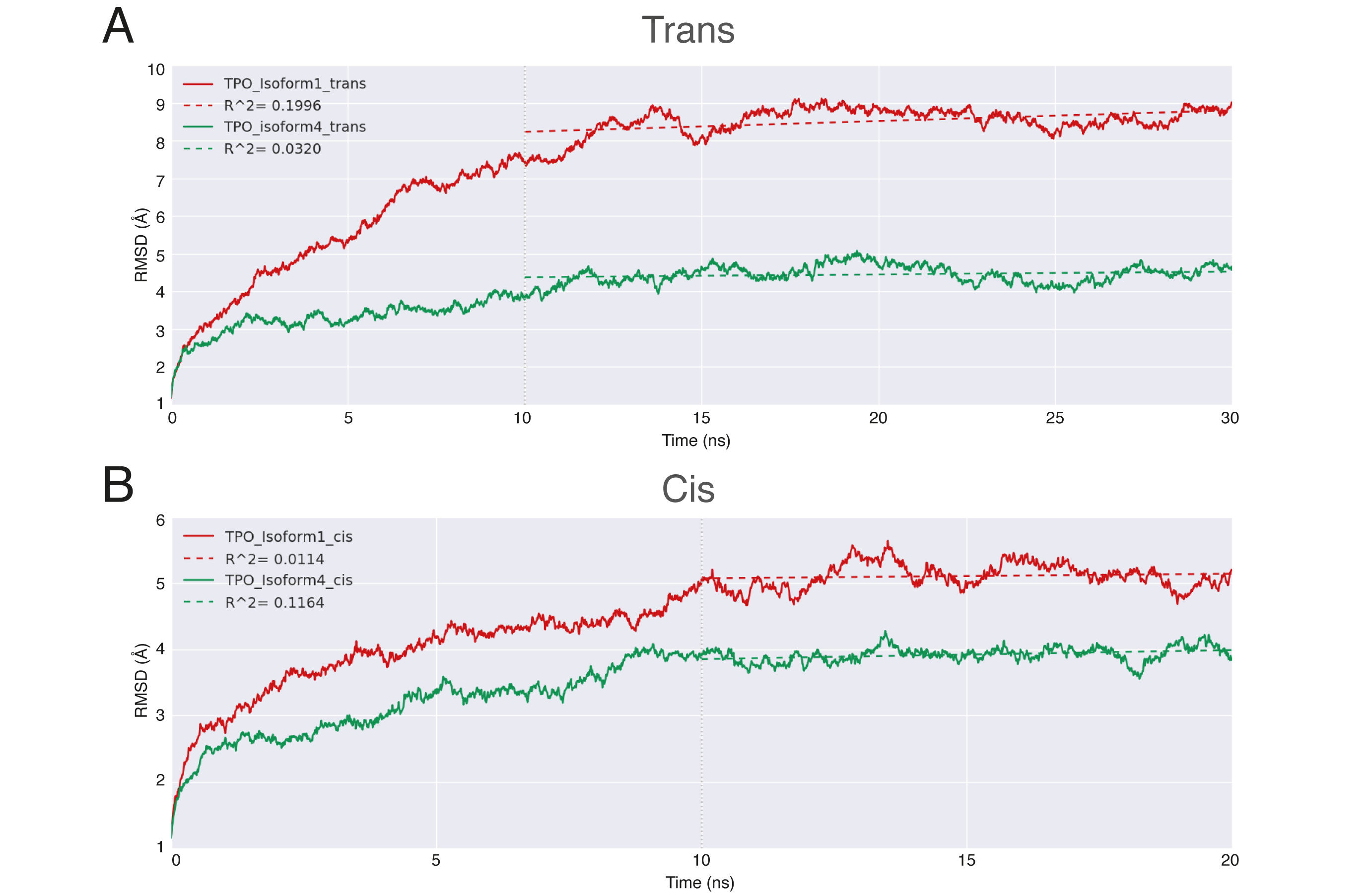

Supplement: S4 Fig — Average Cα backbone RMSDs (n = 1) were found to stabilize after deviating 8.5 Å for isoform1-trans, 5 Å for isoform1-cis, 4.5 Å for isoform 4-trans, and 4 Å for isoform 4-cis. RMSDs indicate that equilibrium is reached after approximately 12 ns for the trans model and 10 ns for the cis model. (PNG) [file pone.0142615.s004.png]

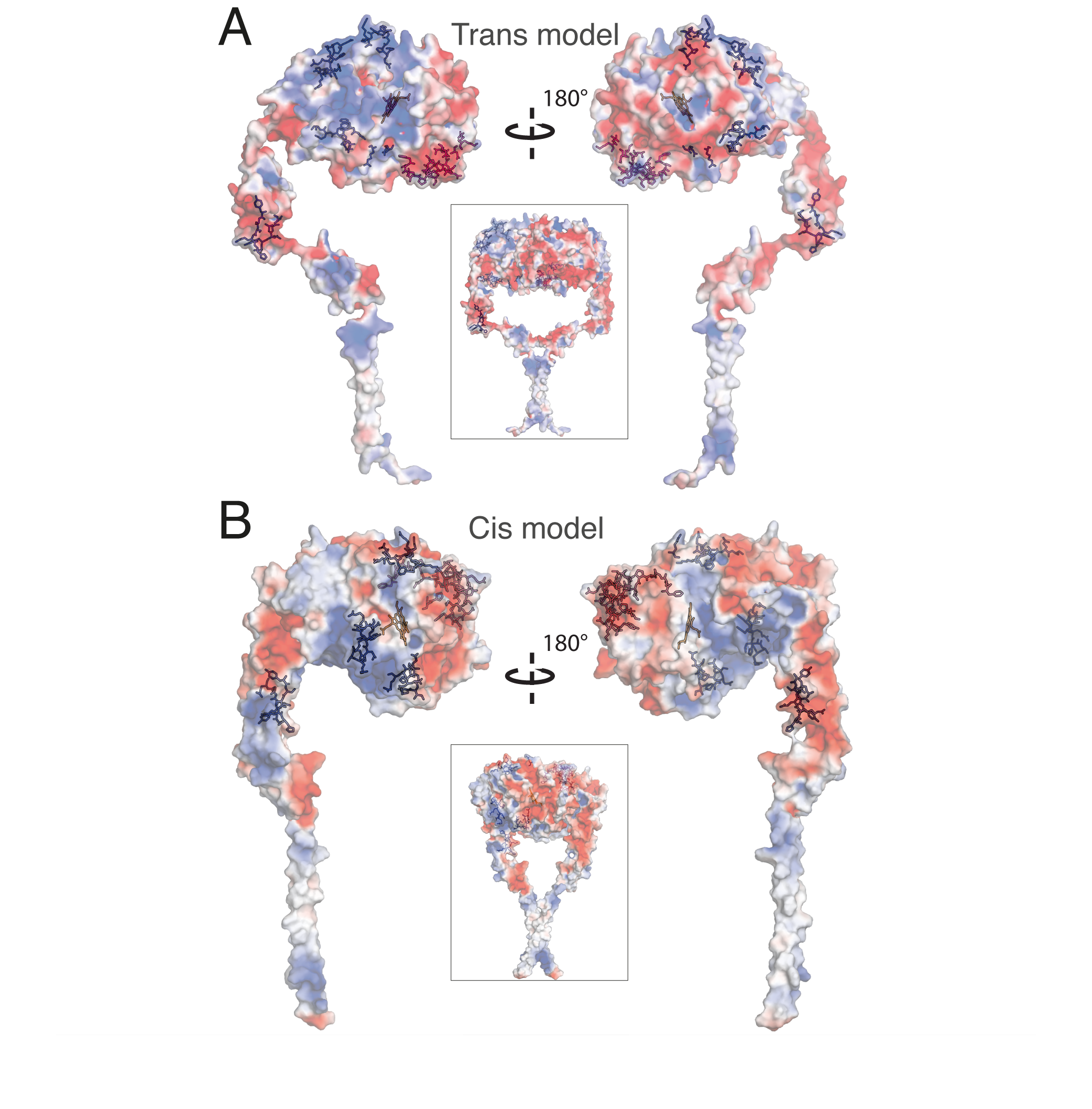

Supplement: S5 Fig — IDR-A is shown in pink sticks, IDR-B is shown in blue sticks and heme is shown in orange. (PNG) [file pone.0142615.s005.png]

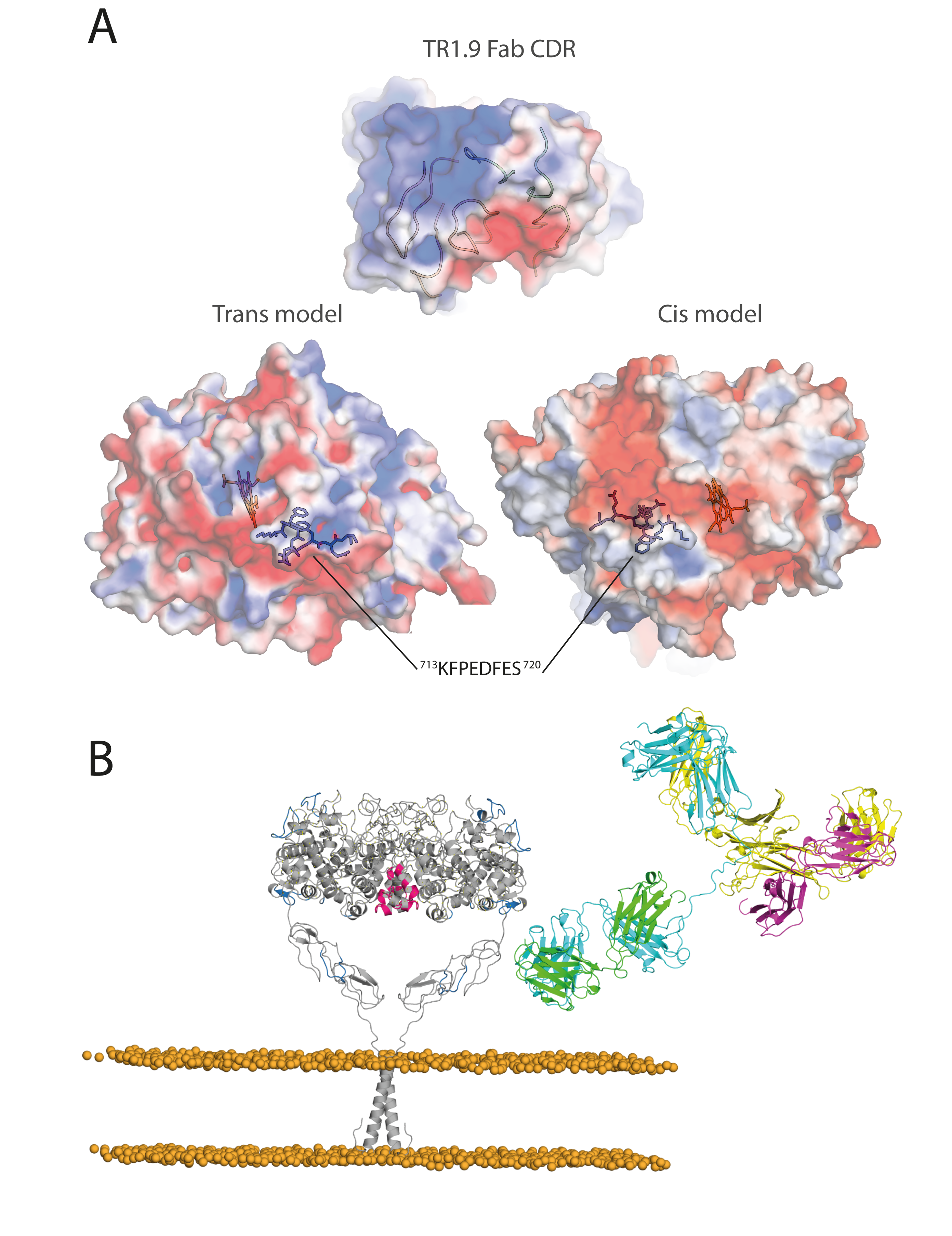

Supplement: S6 Fig — (A) Electrostatic surface potential of the TR1.9 Fab CDR and its respective IDR-A epitope on the trans and cis model. Electrostatic surfaces are contoured at ±3 kT/e (blue is +ve, red is—ve). CDR loops are also shown underneath the transparent molecular surface; (B) Relative size and scale of an IgG molecule and the trans model of TPO, with IDR-A coloured in blue and IDR-B coloured in magenta. This reveals the restricted space available for antibody binding. (PNG) [file pone.0142615.s006.png]
