# Supplementary material for: Modelling of Thyroid Peroxidase Reveals Insights into Its Enzyme Function and Autoantigenicity
Source: PLoS One. 2015 Dec 1;10(12):e0142615. doi: 10.1371/journal.pone.0142615 (PMC4666655; doi:10.1371/journal.pone.0142615)
Supplement: S1 Table — 1Number of unfavorable all-atom steric overlaps ≥ 0.4Å per 1000 atoms. 2MolProbity score combines the clashscore, rotamer, and Ramachandran evaluations into a single score, normalized to be on the same scale as X-ray resolution. 100th percentile is the best among structures of comparable resolution; 0th percentile is the worst. 3Global score of the whole model reflecting the predicted model reliability ranging from 0 to 1. 4Estimate of the "degree of nativeness" of the structural features observed in a model by describing the likelihood that a model is of comparable quality to high-resolution experimental structures. (DOCX) [file pone.0142615.s007.docx]

| **Model** | **MolProbity** | | **QMEAN** **Server** | |
| --- | --- | --- | --- | --- |
|  | Clashscore^1^ | Score^2^ | QMEAN Score^3^ | Z-score^4^ |
| Cis | 0.88 (99^th^ percentile) | 1.71 (89^th^ percentile) | 0.60 | -1.81 |
| Trans | 1.04 (99^th^ percentile) | 1.76 (87^th^ percentile) | 0.59 | -1.87 |
