# Supplementary material for: Modelling of Thyroid Peroxidase Reveals Insights into Its Enzyme Function and Autoantigenicity
Source: PLoS One. 2015 Dec 1;10(12):e0142615. doi: 10.1371/journal.pone.0142615 (PMC4666655; doi:10.1371/journal.pone.0142615)
Supplement: S2 Table — (DOCX) [file pone.0142615.s008.docx]

| **Epitope residues** | **Length** | **Reference^1^** | **Relevant** **antibodies** |
| --- | --- | --- | --- |
| IDR-A |  |  |  |
| 377-386 | 10 | Bresson 2003 [1] | T13 |
| 353-363 | 11 | Bresson 2003, Rebuffat 2006 [1, 2] | T13, ICA1 |
| 713-720 | 8 | Bresson 2003, Bresson 2004 [1, 3] | T13, TR1.9 |
| 713 | 1 | Guo 2001 [4] | TR1.9 |
| 707 | 1 | Dubska 2006 [5] | 126TO10, 126TP1 |
| 225 | 1 | Gora 2004 [6] | 126TP1, 126TO10, 126TP7 |
| 646 | 1 | Dubska 2006 [5] | 126TO10, 126TP1 |
| 766-775 | 10 | Bresson 2003 [1], Estienne 2002 [7] | T13 |
| IDR-B |  |  |  |
| 630 | 1 | Dubska 2006 [5] | 126TP14, 126TP5 |
| 627 | 1 | Gora 2004 [6] | 126TP14, 126TP5, 131TP7 |
| 624 | 1 | Dubska 2006 [5] | 126TP14, 126TP5 |
| 620 | 1 | Dubska 2006 [5] | 126TP14, 126TP5 |
| 611-618 | 8 | Bresson 2005 [8] | TR1.8 |
| 597-604 | 8 | Bresson 2005 [8] | 126TP14, 126TP5, SP1.4, WR1.7 |
